# Stress in Chinese teachers who teach the mathematically gifted: a qualitative perspective

**DOI:** 10.3389/fpsyg.2024.1388236

**Published:** 2024-06-25

**Authors:** Sunzhong Lv, Yijie He, Bin Xiong, Yuchi Wu

**Affiliations:** ^1^College of Mathematics and Physics, Wenzhou University, Wenzhou, China; ^2^School of Mathematical Sciences, East China Normal University, Shanghai, China; ^3^Shanghai Key Laboratory of Pure Mathematics and Mathematical Practice, Shanghai, China

**Keywords:** mathematical olympiad, teachers of the mathematically gifted, pressure, Bronfenbrenner’s ecological system, Chinese teachers

## Abstract

Those who teach mathematically gifted high school students play a key role in both their identification and cultivation. Teachers who teach the Mathematical Olympiad in China work long hours and teach difficult content; they are under a significant amount of work-based pressure, and that is worthy of further study. This study analyzed the sources of stress for 33 Chinese teachers who teach the Mathematical Olympiad, collected data through semi-structured in-depth interviews, and adopted a subject analysis method based on Bronfenbrenner’s ecological system that considers the influences that the complexity of socio-cultural and environment have on individual emotions. It is divided into five structural or environmental systems in which human beings develop, namely the macroscopic, external, meso, micro, and chrono systems. The research results show that the greatest influences on these teachers’ stress come from the mesosystem and microsystem levels, and that the key players are students and school leaders. Educational policy and culture are found to be key factors from the macrosystem. Overall, long-term stress was seen to affect both teachers’ moods and their physical health. Finally, the results offered implications for education policy and school management and suggestions for the cultivation and management of mathematics teachers who teach the gifted. Limitations of the study are discussed, and directions for future research are proposed.

## 1 Introduction

Mathematics is the foundation of scientific and technological innovation; as such, the identification and cultivation of mathematically gifted students is crucial for mathematics education researchers (Singer et al., 2016; Leikin, 2021). The Ministry of Education in China has therefore issued many documents (Lu and Leng, 2020; Du, 2021) that explore diversified admission opportunities and commit to selecting and nurturing outstanding students with excellent comprehensive qualities or top-notch abilities. In China, the five discipline competitions, namely Mathematics, Physics, Chemistry, Biology, and Informatics, alongside the Gaokao, the college entrance examination, have the longest history, the largest number of participants, and the most systematic cultivation mode with Chinese characteristics (Lu and Leng, 2020).

The Mathematical Olympiad plays an important role in identifying and developing teenagers’ mathematical abilities (Kenderov, 2006; Leikin, 2021). Scholars both in China and abroad have carried out relevant studies on mathematical Olympians (Campbell and Walberg, 2010), but the existed studies are not yet sufficient enough (Jung and Lee, 2021). China has consistently achieved impressive results since it first participated in the IMO in 1985 (The IMO Board, 2022). In China, about 50,000 middle school students participate in The National Senior High School Mathematics Competition (NSHSMC) every year. The students who participate in the NSHSMC, should first have their own willingness to take part in, and then need the teachers to recommend and screen, and finally they can obtain the qualification to participate in the NSHSMC if they stand out in the provincial preliminary competition. These students usually have a certain interest and gift in mathematics, and can be regarded as mathematics talents in China. The highest scorers from the provinces can then attend the Chinese Mathematical Olympiad (CMO), which is on par with the IMO in terms of difficulty. Additionally, about 10 teachers in each province can receive the National Excellent Teacher Award from the Chinese Mathematical Society. About 400 students participate in the CMO each year. Ultimately, six students from the national training team are selected to represent China at the IMO.

The past 20 years have seen many IMO winners be awarded the Fields Medal, and many outstanding young talents have appeared in the Chinese Mathematical Olympiad. China’s achievements in the Mathematical Olympiad are dependent on the contributions of front-line teachers, who play a crucial role in training for it (Zha et al., 1996). Overall, teachers play a vital role in supporting gifted students’ inventions and creative achievements (Renzulli, 1968; Croft, 2003). They have the ability to positively influence gifted students, provided that they are aware of their unique educational needs (Porath, 2009). Gifted students’ success is linked to both specialized education and teachers’ support (Campbell et al., 2000; De and Gubbins, 2011). Teachers must therefore be equipped with knowledge regarding the best ways to educate the talented; this includes identifying and nurturing them as well as maintaining an awareness of their social value (Wilhelm and Prado, 2000; Davis et al., 2014). However, despite the level of relevance and importance, there are very few studies on teachers of mathematically gifted students (Karp, 2010; Leikin, 2011; Karsenty, 2014).

A prospective area of research is the qualification of teachers preparing students for mathematics competitions, and not every mathematics teacher can fulfill this task which requires intent, ambition, some other personal qualities, and most importantly, special nontrivial preparation (Bankov, 2022). In China, teachers of mathematically gifted students must not only cultivate basic mathematical skills; they are also in charge of teaching the Mathematical Olympiad (Zha et al., 1996), which is a challenging job. The differences between the work of teachers of mathematically gifted students and regular teachers mainly lie in the following two points. First of all, in terms of work content, regular math teachers only need to be responsible for the teaching work aiming at the college entrance examination. However, besides the Gaokao-oriented math knowledge, the teachers of mathematically gifted students should master knowledge of specialized competition courses as well, such as geometry, algebra, number theory and combinatorics (Nieto-Said and Sánchez-Lamoneda, 2022), which is far more difficult than knowledge of the Gaokao. Secondly, in terms of working hours, on weekends and holidays, regular teachers can rest, while teachers of mathematically gifted students still need to work. They are required to provide additional tutoring for students or to take students to summer camps, winter camps or other Mathematical Olympiad activities. Therefore, owing to the more complicated nature of work and the heavier tasks, teachers of mathematically gifted students consume more time and efforts in work than regular teachers, but their income from work is not high (Sunzhong et al., 2022). Due to its particularities, teachers of mathematically gifted students shoulder a lot of work-related pressure and psychological burden. They often experience anxiety and poor physical health, which can lead to burnout and resignation intention (Liu and Onwuegbuzie, 2012), and even affect students’ learning. Therefore, educators and educational administrative departments should prioritize lessening the pressure on teachers and create a better working environment. However, little attention has been paid to the details of teachers’ work in nurturing mathematically gifted students and their negative feelings regarding that work. Therefore, studying the work-based pressure that they experience and determining its reasons are significant for both the cultivation of gifted students and the development of their teachers. Accordingly, the research questions are as follows:

1. What are some factors that lead to stress for teachers of mathematically gifted students?
2. How do these factors impact teachers’ personal and professional lives?

## 2 Literature review

### 2.1 Teachers’ stress

The definition of stress changes significantly across various scientific disciplines. Stress involves both psychological and physiological changes that can be observed and measured; some examples include measurements via brain activation, cellular mechanisms, and people’s subjective experiences (Von Dawans et al., 2021). Teachers’ stress can be defined as a response of negative affect; workplace demands can threaten either their self-esteem or well-being. At the same time, coping mechanisms are activated to reduce the perceived threat (Kyriacou and Sutcliffe, 1978). As a result, teachers’ workplace stress has been defined through unpleasant, negative emotions, such as anger, tension, frustration, or depression (Kyriacou, 2001).

From the perspective of the teachers themselves, the daily demands of their work and their busy lives are their main sources of stress (Ferguson et al., 2012; Skaalvik and Skaalvik, 2015). Teachers have a limited amount of time to complete class preparations, create necessary materials, and communicate with students, which can make them overtired (Ferguson et al., 2012; Skaalvik and Skaalvik, 2015). Overall, individuals are more likely to experience conflicts between work and family life when faced with heavier workloads (Liu and Cheung, 2015). High levels of stress have been shown to undermine one’s concentration, empathy, memory, and sleep (Von Dawans et al., 2021). In the case of math teachers, math-based anxiety is another key factor, especially for new teachers (Bekdemir, 2010).

Teachers’ pressure mainly comes from students’ behavior (Shen et al., 2015; Bottiani et al., 2019), which is an important factor causing teachers’ anxiety (Ferguson et al., 2012; Skaalvik and Skaalvik, 2015). Teachers may also experience stress based on the number of students in their class (Herman et al., 2018; Bottiani et al., 2019). The schools themselves can also affect teachers’ stress in several ways, including testing (Nathaniel et al., 2016; Ryan et al., 2017) and interpersonal relationships with school leaders and colleagues (Skaalvik and Skaalvik, 2015; Bottiani et al., 2019). According to various studies on test-based accountability systems, these types of evaluations of teachers lead to them experiencing increased pressure, which, in turn, leads to increased outflow. Teachers may also resign due to a lack of support from and pressure applied by school leaders (Ryan et al., 2017). In addition, social factors such as national education policies (Yin et al., 2011), tutoring institutions (Liang, 2014; Zhang et al., 2022), and competition among schools (Andersen and Serritzlew, 2007; Kai, 2012) can cause stress and anxiety in teachers.

Teachers in China have a particular historical background that is worth noting. Chinese students may enter universities via the Gaokao; only a small number are recommended directly to universities through the Mathematical Olympiad (Xiong and Jiang, 2021). Chinese students also work very hard and have a strong internal and external motivation to learn (Leung, 2001; Wong et al., 2012); scholastic success or failure has the ability to affect a family (Li, 2002).

The pressure involved in students entering universities means that Chinese teachers need to provide extra tutoring to students in their off hours, which greatly increases their workload (Yang et al., 2015). Moreover, Chinese classes usually have a large number of students, but teachers are poorly paid for their work (Wu et al., 2006; Wang et al., 2009). Some empirical studies have been conducted on the work-based stress of teachers in China (Wu et al., 2006; Wang et al., 2009; Yang et al., 2015) and others have compared the stress levels of teachers in China with those of teachers in western countries (Liu and Onwuegbuzie, 2012, 2014). In addition, the huge gap in educational resources between urban and rural areas leads to increased pressure on Chinese teachers as compared to other developed countries (Liu and Onwuegbuzie, 2012).

### 2.2 Conceptions of giftedness and the characteristics of gifted students

There are many competing conceptualizations of giftedness (Sternberg and Davidson, 2005) that emphasize multiple categories, types, and components of giftedness or intelligence. Interdisciplinary exploration must be undertaken to better understand giftedness. Despite all the differences in points of view, there are some commonalities across these diverse viewpoints. Sternberg and Ambrose (2021) summarized 10 uniform points of agreement on giftedness and talent; these included motivation, passion, and attitude. The characteristics of gifted children are tied to how they are identified as such; Davis et al. (2014) offered a collection of recurrent positive and negative characteristics of gifted students.

Conventional perspectives on intelligence and giftedness, spanning from general factors and associated methodologies (Spearman, 1904; Cattell, 1987; Jensen, 1998) to more distinct models (Thurstone, 1938; Guilford, 1967; Carroll, 1993; Feldhusen, 1998), perceive these constructs as inherent to the individual. While many of these theories acknowledge the influence of the environment on intelligence development, the primary focus remains on the individual as the locus of control and the primary point of interest. In contrast, a few contemporary approaches incorporate the role of environment when discussing intellectual ability and talent. Gardner (1983) places significant emphasis on cultural context across various applications of his Theory of Multiple Intelligences. Renzulli’s (1978) well-recognized Theory of Giftedness introduces the concept of the three-ring conception, which highlights the interplay among above-average ability, creativity, and task commitment.

Mathematical giftedness is sometimes seen as a specific aspect or type of giftedness; its domain-specificity always implies a collection of certain mathematical abilities and personal qualities (Singer et al., 2016). Mathematics competitions are essential for identifying, challenging, and enhancing gifted students’ mathematical expertise. Examining the students’ views on their experiences with these competitions may help evaluate their effectiveness on both the development of contestants’ mathematics abilities and their future career choices (Campbell et al., 2000; Taylor, 2017). A systematic review of existing surveys concerning Mathematical Olympiad winners maintained positive attitudes toward their experiences (He et al., 2022).

### 2.3 Study of teachers of mathematically gifted students

The study of teachers who teach gifted students has attracted many researchers’ interest and attention. Existing studies primarily focus on teachers’ conceptions of giftedness, competence characteristics, and professional development. Based on a systematic review of related studies, Whitlock and DuCette (1989) indicated that the outstanding characteristics of successful teachers of the gifted are their enthusiasm and self-confidence, strong achievement orientation, and previous recognition as outstanding educators. Feldhusen (1997) summarized the characteristics and competencies of successful teachers of gifted and talented students while VanTassel-Baska (2005) found that the ideal profile of a teacher for the gifted included competencies such as mastery of disciplinary content knowledge, positive interaction with students, and utilization of diverse teaching strategies. Studies have also shown that many teachers believe that gifted students tend to show less social adjustment than students with average abilities (Baudson and Preckel, 2016).

Studies on teachers of mathematically gifted students specifically are scarce (Singer et al., 2016). In an earlier study, Stanley (1975) referred to the competencies required of teachers who teach mathematically gifted students from grade 4 to grade 12. Karp (2010) interviewed two teachers in Russia, studied their teaching characteristics, and discussed the influence of their mathematically gifted students on them. Leikin (2011) analyzed teaching characteristics and summarized the qualities a teacher must have in order to cultivate gifted mathematics students. Shayshon et al. (2014) compared mathematics teachers’ perception of the mathematically gifted in three countries, and found that their perceptions can be affected by both the different learning backgrounds of their students and experiences teaching classes of different sizes. Karsenty (2014) conducted a study of a 2-year mathematics teacher’s training course, which showed that a strong mathematics background and experience with communicating with mathematically gifted students helped trainees successfully complete the course. Hoth et al. (2016) found that teachers who had difficulty with logical reasoning and understanding mathematical structures also had difficulty identifying mathematically gifted students. Sunzhong et al. (2022) takes four seasoned and outstanding Chinese teachers as cases, and uses the thematic analysis method based on Herzberg’s two-factor theory to analyze why they can persist in the field of Mathematical Olympiad teaching for more than 20 years. Baker et al. (2022) find that teachers view their students’ participation in mathematics competitions as being important, with the biggest barrier to participation being that there are not enough competitions at appropriate levels available in Africa. Teachers are unlikely to put effort into supporting competitions that are not curriculum based. Also, many rural schools across Africa do not have strong internet connections, and the cost of data bundles can restrict the teachers’ ability to get online.

Studies with a focus on the characteristics and competencies of Chinese teachers of mathematically gifted students are limited (Chan, 2001, 2011; Cheung and Hui, 2011), and little research has been found on their work-based stress and emotional wellbeing.

## 3 Theoretical framework

Bronfenbrenner (2005) proposed an ecological system framework that considers the complexity of socio-cultural and environmental influences on individual emotions. He identified five structural or environmental systems in which human beings develop, namely the macroscopic, external, meso, micro, and chrono systems. Within this framework, types of interactions range from close to distant (Tissington, 2008).

Researches on teachers’ emotional development based on ecological framework mostly adopt qualitative research design such as case study and narrative inquiry (Chen, 2017, 2020; Carroll et al., 2021). Chen (2017) found that, in the five ecosystems, different types of emotions decrease as the distance from the teachers increase. In addition, Chen (2020) utilized a mixed method approach to further investigate the emotions of 1,492 primary school teachers in China. Evidence from both qualitative and quantitative data suggested that high levels of emotional intensity were apparent at the microsystem level. Carroll et al. (2021) explored 74 teachers’ perceptions of their major sources of stress at work and surveyed the methods they used to help alleviate that stress. The ecological system theory revealed a series of systematic, organizational, relational, and intrapersonal stressors that are the driving forces behind teachers’ stress in the workplace.

The microsystem includes settings where people live or someone people directly interact with but he may be changing and developing (Bronfenbrenner, 2005). Exploring teachers’ interactions at the microsystem level will clarify how they communicate and feel when interacting with stakeholders in both the classroom and at home (Chen, 2020). For the teachers in this study, their interactions with family members and the teaching tasks were included in this system. Long working hours and complex mathematical problems both affect the lives and work of teachers of the mathematically gifted (Sunzhong et al., 2022). The mesosystem involves the relationships and processes that occur between two or more changing, developing settings or people (Bronfenbrenner, 2005). In the teacher ecological environment, teachers frequently sought professional collaboration on a broader layer of the environment for development, which constituted a mesosystem (Tissington, 2008). In this study, the mesosystem for teachers included links with students, colleagues, and school leaders. Those persons who participate actively in the settings influence them greatly within this system. The exosystem exists between two or more settings, at least one of which does not change or evolve (Bronfenbrenner, 2005); in short, the exosystem is a wider social system, such as a parent-teacher organization or government agency. The exosystem for the teachers in this study was primarily comprised of students’ parents and organizations which include other high schools and tutoring institutions. The attitudes of students’ parents towards mathematics competitions, inter-school competition, and the interventions of training institutions on in-school teaching work all affect the work of teachers of the mathematically gifted (Sunzhong et al., 2022). The macrosystem is defined as a pattern of ideology and a social institution that can illustrate a particular culture or subculture (Bronfenbrenner, 2005); it represents the societal environment in which teachers work in accordance with particular norms, values, regulations, and policies (Cross and Hong, 2012). The teachers’ working lives in this study were primarily influenced by cultural and political elements, which were the most visible manifestations of the macrosystem. In China, college entrance policies related to mathematics competitions also change over time. Lastly, the chronosystem allows one to identify the effects of their prior life experiences, either singly or sequentially, on their subsequent development (Bronfenbrenner, 2005). Teachers are always experiencing different life events with different students at different times, which inevitably change them (Chen, 2020). As for teachers in this research, what change over time are their mood and their health.

**Figure 1:**
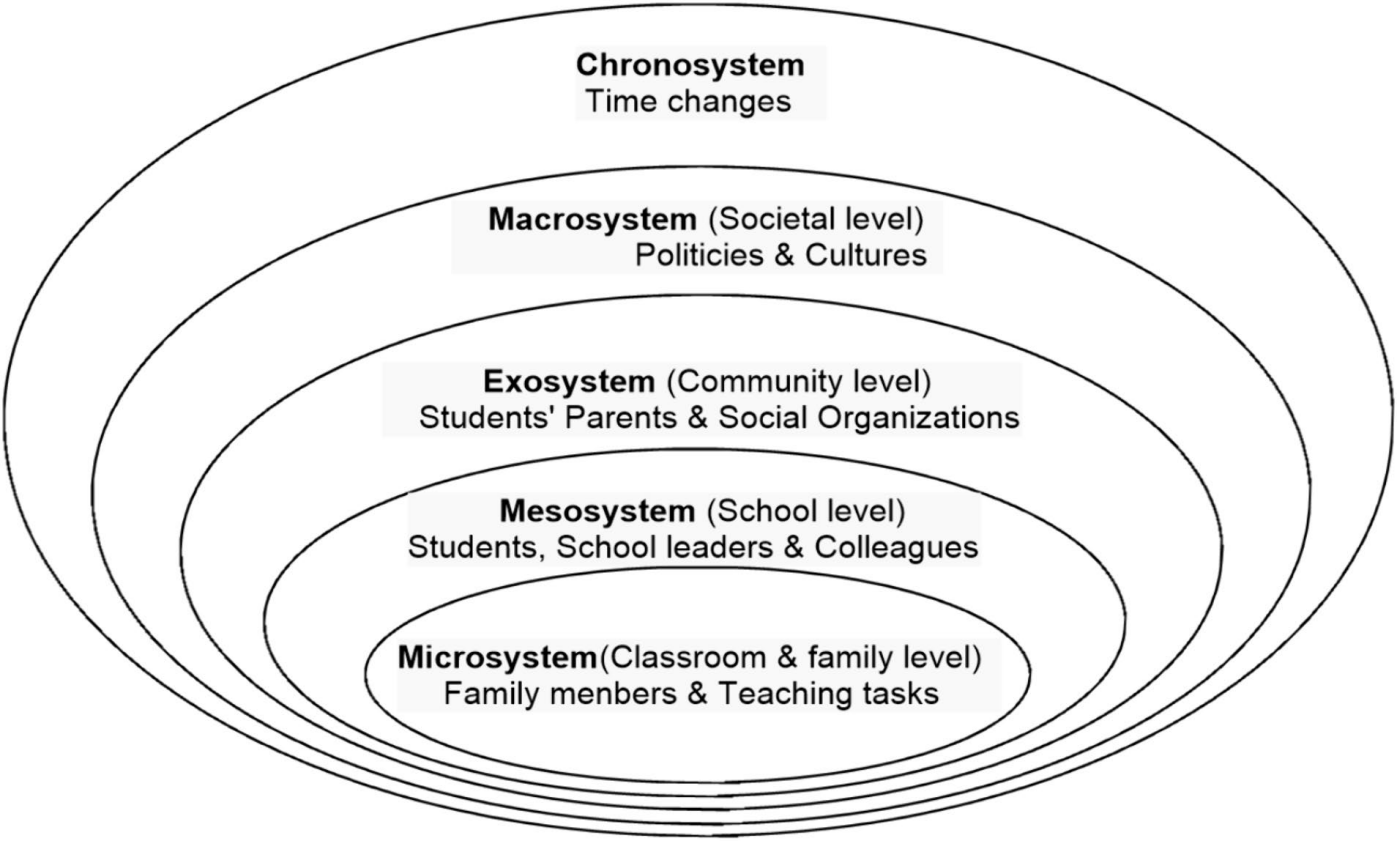
A revised framework adapted from Bronfenbrenner (Chen, 2020).

Teachers’ stress is complex and multiterminal, involving dynamic interactions between internal and organizational factors. Therefore, ecological system models like Bronfenbrenner (2005), which consider multiple influence contexts (e.g., systemic, organizational, relational, and internal stressors) may be a useful means for better understanding the competitive and dynamic factors that shape teachers’ work-related stress. Based on the model proposed by Chen (2020), this study systematically investigated and summarized the ecological factors involved in teachers’ emotions (see Figure 1). Using the five ecological systems, we aim to conduct a systematic, comprehensive investigation of the stress experienced by 33 high school mathematics teachers in China during their teaching lives and the factors that affect that stress.

## 4 Research design

### 4.1 Participants in the study

Prior to this study, the research team conducted online surveys to investigate teachers’ sources of mathematical Olympiad knowledge and their emotions towards mathematically gifted students. These studies were conducted separately. Some teachers voluntarily provided their contact information for further surveys, and the 33 participants in this study were recruited from this group. Only one of the participants was female; this is in accordance with Chinese standards, where most teachers who teach the Mathematical Olympiad are male. A purposive sampling approach was taken to recruit participants for the current study based on their judgments of their own possession of the particular characteristics being sought (Cohen et al., 2011). The participants were all mathematics teachers who also teach the Mathematical Olympiad. They all serve the frontline of education. In school they need to undertake the teaching tasks of both the Gaokao and the Mathematical Olympiad, which means they not only need to help all the students develop the mathematical foundation, but also need to elevate the mathematical ability of gifted students.

In terms of professional titles, there were 14 associate senior teachers and one senior teacher (Tan, 2013). In terms of educational background, 13 teachers had master’s degrees. In terms of age, the youngest teacher was 27 years old and the oldest was 57; the average age of the participants was about 38.2 years old. In terms of their experience teaching the Mathematical Olympiad, the newest had taught it for 3 years and the most seasoned had taught it for 30. Participants had been teaching for an average of 11.5 years. Twenty-four teachers had previously been awarded the National Excellent Teacher, five had been awarded the distinction of Senior Coach of the China Mathematics Olympiad, 18 had taught students who achieved bronze medals and above in the CMO, and two had trained who achieved gold medals in the IMO.

### 4.2 Data collection

This research was conducted in accordance with the ethical standards of and checked by the Committee on Human Research Protection of East China Normal University. After obtaining participants’ consent, we scheduled the interview time. Then, we conducted an online semi-structured interview with each participant individually.

This research was conducted in accordance with the ethical standards of and checked by the Committee on Human Research Protection of East China Normal University. After obtaining participants’ consent, we scheduled the interview time. Prior to the interviews, we clearly explained the purpose of the study, what would be discussed, and any potential risks or discomfort that participants might experience. Participants could have the option to withdraw their consent at any time without negative consequences. Then, we conducted an online semi-structured interview with each participant individually. Each in-depth interview lasted for 40–90 min. During the interview process, we also made adjustments to the preset questions, paraphrased, or asked further questions as needed to obtain as much information as possible. Then, through recording and audio transcription, the interviews were converted into text and research notes were taken; any names or specific locations mentioned during the interviews were anonymized. For ease of processing, we gave numbers A_1_ to A_33_ to the 33 teachers.

This study used a semi-structured interview format with questions as follows: ① When did you decide to start teaching the Mathematical Olympiad, and why did you choose to do so? ② Please talk about your daily work as a teacher for the mathematically gifted, and what abilities or qualities do you find most essential in this work? ③ In the process of teaching, what key factors have influenced your willingness to go in for teaching the Mathematical Olympiad? ④ Please share some reasons why some math teachers have either chosen to teach or stop teaching the Mathematical Olympiad. This study draws on Kyriacou’s (2001) research, where teachers’ workplace stress is defined as the experience of unpleasant, negative emotions such as anger, tension, frustration, or depression. These emotions can be characterized as a response of negative effects. Internal factors within the school environment, such as math-based anxiety, students’ behavior, school leaders, and the daily demands of teachers’ work and busy lives, all impact teachers’ job performance (Bekdemir, 2010; Ferguson et al., 2012; Shen et al., 2015; Skaalvik and Skaalvik, 2015; Ryan et al., 2017; Bottiani et al., 2019). Additionally, social factors such as national education policies, tutoring institutions, and competition among schools also influence teachers’ work (Andersen and Serritzlew, 2007; Yin et al., 2011; Kai, 2012; Liang, 2014; Zhang et al., 2022). Although the four questions are not directly related to teachers’ stress, during the interview we further asked some questions related to teachers’ work stress according to their instant answers. For example, what difficulties teachers face when starting to engage in teaching the Mathematical Olympiad, how they deal with problems they cannot understand, the negative emotions produced in daily work, the relationship between teachers and leaders, colleagues, parents, and students, the confusion they encounter in improving their teaching ability of mathematics competition, the negative emotions that affect teachers engaged in teaching the Mathematical Olympiad, experiences that frustrate teachers’ enthusiasm in teaching, the reasons why they give up teaching the Mathematical Olympiad and so on. Of course, because the interview data is huge, and what were mentioned by teachers are not restricted to stress, we extracted and coded the topics concerning stress.

### 4.3 Data analysis

In order to create a comprehensive analysis of the research problem, this study used a qualitative approach by conducting individual interviews. Thematic analysis (Braun and Clarke, 2006) and three-level coding (Corbin and Strauss, 2008) were also used to analyze interview data with Nvivo 12 as an assisting analysis tool (see Table 1). The results provided us with 334 nodes (nodes are the units used by Nvivo 12). We then divided these nodes into 10 factors (with 26 sub-factors) based on the research questions. Finally, using selective coding, we categorized the 10 factors into five themes based on Bronfenbrenner’s (2005) ecological system. What should be noted is that if some sentences belong to multiple dimensions, we counted them once in each corresponding dimension. That is to say, a sentence can subordinate to multiple dimensions. In the data analysis stage, one researcher independently encoded the text twice, compared the results, and then discussed them with the other researchers in order to ensure the reliability and validity of the coding.

**Table 1:** A sample of data coding steps.

Original material	Open coding	Axial coding	Selective coding
7: School leaders will put a lot of pressure on teachers, pushing them to ensure that more students get better results in competitions, which is similar to companies’ kpi indicators.	School leaders overly emphasize scores	School leaders	Mesosystem
13:Some students’ parents do not support Mathematical Olympiad because they think participating will impact their children’s performance on their college entrance examinations.	Students’ parents do not support	Students’ parents	Exosystem

## 5 Qualitative analysis

### 5.1 Microsystem

Overall, 31 participants made 91 negative comments involving their microsystems, particularly their family members and teaching responsibilities. Although teachers’ family members are in the more intimate relationships with them, students are their main service objects and primary influences on their teaching. Teachers’ family members were seen to be related to negative emotions such as guilt and annoyance while teaching tasks led to other negative emotions, including pressure, anxiety, pain, confusion, frustration, exhaustion, worry, self-doubt, and helplessness.

#### 5.1.1 Family members

Due to increased work commitments, teachers who teach the Mathematical Olympiad have little time to spend with their family members. Some of their family members occasionally complain that they have no time, especially on weekends and holidays. Some teachers feel guilty about having no time care for their families, particularly their elderly relatives and children. They are also troubled by the difficulty of maintaining a work-life balance.

A_14_: *My wife sometimes complains that I have almost no free time on the weekends. Instead, I go to work and teach the Mathematical Olympiad. Sunday mornings may be my only free time during the week.*

Teachers work long hours, which causes them less time to accompany their families and no time to rest all year round. Besides, squeezed personal life, tiredness and anxiety are also results of it. Because of teaching the Mathematical Olympiad, as the non-regular class, is usually arranged during resting time such as weekends, holidays, and weekdays’ evenings. So teachers do not have much time to spend with their families.

#### 5.1.2 Teaching tasks

It seems obvious that teachers who would want to teach Mathematical Olympiad would also be interested in teaching and working with gifted students. Since most Mathematical Olympians have extraordinary talents, teachers have higher expectations for them and are willing to provide more assistance in developing these students both physically and mentally so as to cultivate top-notch mathematical talent for China. However, the level of these students’ talents means that some of them are better at solving mathematics problems than their teachers by the end of their first year of high school. Inevitably, teachers experience pressure because they may be incapable of providing professional teaching to these students; they may even have doubts regarding whether their teaching is helpful.

A_18_: *When teaching the gifted students, I feel very anxious. I question whether my teaching is a waste of their time and wonder if my help is enough for them to improve.*

In addition to the long hours, the workload of teachers of mathematically gifted students is also substantial. In addition to their regular teaching, they must also create and plan their lessons, manage their students on a day-to-day basis, and plan field trips. These are all part of a teacher’s daily work with mathematically gifted students. In terms of problem solving and lesson preparation, teaching the Mathematical Olympiad is difficult and complicated. Some teachers cannot solve the problems themselves, which causes pain and frustration. Moreover, in China, there is no unified teaching material for the Mathematical Olympiad; teachers must collect the materials by themselves, which puts great pressure on them and may make them anxious and confused.

A_5_: *Teaching the Mathematical Olympiad is extremely arduous for me because we need to prepare mathematics problems for the students. However, finding those materials and choosing the types of problems always costs me a lot of time. When the students complete their work, we, of course, offer feedback. However, we cannot offer feedback if we are unable to complete the problems ourselves, which causes anxiety and, ironically, negatively affects our ability to solve the problems.*

It should be emphasized that, due to the difficulty of the Mathematical Olympiad, teachers often encounter math problems that they themselves cannot solve but still have to explain to students. Sometimes they may even need to seek help from the students. At the same time, due to the specifics of the Mathematical Olympiad and the lack of unified teaching materials and instructions, some teachers feel a lack of clarity on both the content and the direction of the tests. Given the lack of guidance and the fact that some students are better than them when it comes to solving mathematics problems, it’s natural for them to doubt the effectiveness of their teaching.

A_24_: *When teaching, I may give up entirely if I do not understand the problem. Other times, I force myself to get through the problem and show the answers to the students, because they may understand something that I do not.*

These teachers are under tremendous pressure; the difficulty of the problems and the talents of their students mean that they have to constantly push themselves to improve in addition to their regular teaching tasks.

A_16_: *I have to practice a lot of math problems, figure out each of them, and then teach them to students. Problems for the Mathematical Olympiad are difficult, particularly combinatorics. After studying the combinatorics problems myself, in addition to my regular workload, I feel stressed. If the geometry and algebra problems are exceedingly difficult, I may not solve them at all.*

### 5.2 Mesosystem

Overall, 32 participants made 118 negative comments involving their mesosystems, particularly their students, administrators, and colleagues. School leaders and colleagues are key parts of teachers’ work; on one hand, wise and effective leadership can reduce the workload on teachers and cooperative colleagues can improve efficiency. On the other hand, they can also cause problems for teachers, including stress, pain and disappointment caused by leaders and disappointment, loneliness, and depression can be caused by colleagues. In addition to students’ classroom performance, their learning behaviors after class can also affect teachers, especially in terms of academic performance and learning attitude; issues in this regard can make teachers can feel regretful, disappointed, depressed, and guilty.

#### 5.2.1 Students

The Mathematical Olympiad itself is also difficult and strenuous in a way that is not suitable for all students; some students will be resistant to it from the very beginning, and some will quit halfway, causing a lot of trouble for teachers, who may be worried and frustrated if some students give up.

A_4_: *Many students cannot stick it out until the competition. They may feel that they are not suitable for it and just give up.*

Many teachers of mathematically gifted students hope for their students to achieve outstanding and officially recognized results because these excellent performances can bring teachers rewards, honors, and recognition of their own. However, even those students who persevere may not achieve ideal results; some may even perform worse than usual. Bad results bring no rewards to teachers, and may even result in negative evaluations while also discouraging students and making teachers feel guilty.

A_30_: *Once, I taught just three students. One of them received the second prize at the provincial level, but the other two got no prizes. The best one I taught got nothing. At that moment, I felt so guilty that I could not forgive myself and nearly gave up teaching the Mathematical Olympiad.*

In addition to these feelings of regret, disappointment, and self-accusation caused by students’ unsatisfactory results, teachers also suffer from pressure, anxiety and worry about whether their students will get good scores.

A_7_: *As the competition approaches, if you want your students to perform well, you should keep them working on mathematics problems; however, you must also consider their moods. Sometimes it’s hard to make students practice more without negatively affecting their moods; teachers often take on their anxiety in that case. I definitely have a relatively high level of anxiety near the time of competition and just before results are released.*

It’s also difficult for students to have the proper mindset during the process of learning the Mathematical Olympiad. For example, some may be overly persistent with the Mathematical Olympiad but neglect their regular courses, others may end up with some psychological illnesses, and still others may be excessively ambitious, clinging to their goals of either getting into better universities or proving their abilities.

A_2_: *He may be relatively ambitious, and he wants to prove himself by learning the Mathematical Olympiad because someone told him it is the most difficult. There are so many students like him every year.*

Students’ learning attitudes, behavior and academic performance can affect teachers’ and cause them stress. Teachers of mathematically gifted students invest a lot of time and energy into their teaching, and so they have high expectations for their students. Some teachers stake their reputations on the future successes of their students. Their students’ scores determine the attitudes of their leaders and colleagues towards them, and their students’ achievements are directly linked to their financial interests, including bonuses and career advancement.

#### 5.2.2 School leaders

School leaders play a key role in influencing teachers’ working moods. They are in charge of planning and implementing courses, inviting experts to speak, and arranging for students to participate in Mathematical Olympiad activities. Their opinions determine how many teaching hours will be allotted for the Mathematical Olympiad. Different leaders have different educational ideas; those who value students’ all-round development may limit the time that students have to learn the Mathematical Olympiad while those who focus on students’ achievements may put pressure on teachers to make sure the students reach certain goals. School leaders may also overstep their boundaries by overly intervening in classes, which upsets teachers.

A_22_: *The influence of the school leaders is still based on students’ scores in the Mathematical Olympiad. If students achieve good results, leaders will have a positive attitude toward us. If the opposite occurs, leaders will doubt us, thus undermining our enthusiasm.*

A_31_: *The situation is that school leaders overly focus on the scores of the tests rather than the development of the students.*

There are two reasons why school leaders attach such importance to the Mathematical Olympiad. Firstly, there are intersections between the content of the Mathematical Olympiad and that of the Gaokao; as such, success in the Mathematical Olympiad could imply success in the Gaokao. Mathematics is also an extremely important subject in autonomous enrollment program of universities. Secondly, students’ achievements in the Mathematical Olympiad are directly linked to their college admissions; universities, such as Tsinghua University, Peking University, and Fudan University attach great importance to students’ scores. In China, the Gaokao is the main way for students to enter universities and their scores are the most important factor. However, School leaders may struggle in finding the balance between prioritizing the Mathematical Olympiad and the Gaokao.

#### 5.2.3 Colleagues

Colleagues are adjustable factors that play a marginal role in teaching the Mathematical Olympiad. Many teachers said that they do not have a professional teaching team for the Mathematical Olympiad or colleagues with whom they can discuss the problems. Due to the limited learning time, the long-term study of the Mathematical Olympiad will affect students’ performance in other subjects; therefore, those teachers may not respect the Mathematical Olympiad, which can create conflict.

A_1_: *The teaching objectives of teachers who teach the Mathematical Olympiad are in conflict with teachers who teach ordinary subjects, which means they compete for teaching time. Especially in elite schools, this kind of conflict may be more obvious and may even need to be resolved by school leaders.*

In addition, there are five discipline competitions in elite Chinese high schools. In many schools, students are free to study any subject they like; some even choose to compete in multiple discipline competitions. However, because of limited number of students who can achieve the best results, the teachers of those five competitions also find themselves competing; some may feel disappointed and depressed if they fail to court the best students.

### 5.3 Exosystem

Overall, 22 participants made 56 negative comments involving their exosystem, particularly their students’ parents and the various organizations that they interact with. Family education is an important part of children’s studies, and parents play a vital role in it. Tutoring institutions, as the after-school supplement for students’ study, towards which teachers have different attitudes. Competition among schools amplifies teachers’ work-related stress; their helplessness, disappointment, and frustration can be caused by students’ parents, while their anxiety and pressure can be caused by organizations.

#### 5.3.1 Students’ parents

Students cannot learn without their parents’ support. Parents often have their own opinions regarding the Mathematical Olympiad and can also provide important support to their children, especially when they go to tutoring institutions or participate in Mathematical Olympiad activities. Parents accompany children and pay for transportation and training, which is a considerable expense for some families. Additionally, some parents do not understand the educational value of the Mathematical Olympiad and even discourage children from participating. Under this circumstance, teachers are left feeling helpless, disappointed, and frustrated.

A_11_: *Parents only support the Gaokao. In fact, they are achievement-oriented. If the Mathematical Olympiad can help their children improve in the Gaokao, they will support it; they may also persuade their children to quit, which has happened before.*

The main reason why parents do not support the Mathematical Olympiad is that they feel that it is not in line with their goal of their children entering university. They are results-oriented and do not pay attention to the process of education itself; it also costs them a lot of money. A less than ideal competition result will result in parental complaints.

#### 5.3.2 Organizations

Off-campus education and training, also known as shadow education, is a kind of educational activity organized by some social tutoring institutions, other than school education. It features personalization, differentiation, diversification and complementarity, which can make up for the shortage of schooling. However, there are also problems, such as unclear position, non-standard operation, non-conforming teaching content. To ensure the benign development of education, the government of China adopts the strategy of supervision and control against the overflowing off-campus education and training (Zhu, 2022). Tutoring institutions also influence the way in which students learn. Most students have studied at tutoring institutions, and some use them frequently. However, teachers have different views on them. Some feel that tutoring institutions can provide essential supplementary time for studying for the Mathematical Olympiad while others disagree. They think tutoring institutions do not take inherent abilities of the students into account, believing that they cost students a lot of money and time with few results.

A_1_: *These tutoring institutions, to some extent, are the opposite of the learning in schools because students need to spare some of their limited time for them.*

Tutoring institutions have a profound impact on students, schools, families and educational order. The market attribute of off-campus tutoring institutions leads to excessive involvement of capital, hence violating the public welfare attribute of education and destroying the normal ecology of education. One thing to note is that the increasing family expenditures for education caused by participation in tutoring institutions will make some parents opposed to their children’s participation in learning the Mathematical Olympiad.

As the Mathematical Olympiad has become more popular, more schools participate, and teaching it has become more specialized. The flexible systems of private schools, in terms of enrollment, for example, attract higher-quality teachers, resulting in fierce local competition.

A_8_: *As compared to before 2008, the competition is too fierce now. With the integrating of some teachers from other provinces, several private schools in our province have become more fierce competition.*

At the same time, high schools have met severe challenges in enrolling new students. Since the number of gifted students is limited, competition for them extremely fierce, and some teachers say that private schools put a great deal of pressure and a sense of urgency on themselves.

### 5.4 Macrosystem

Overall, 19 participants made 43 negative comments involving their macrosystem, mainly concerning policies. China attaches great importance to the finding and cultivation of top-notch talent. As such, the Ministry of Education has issued a number of relevant policies intended to assist in cultivating those students who are talented in basic subjects. However, these policies also affect students’ studies and teachers’ work overall, leaving some teachers worried, dissatisfied, and helpless.

#### 5.4.1 Policies

Students’ admission to universities through participating in Mathematical Olympiad can be directly affected by education policies. More importantly, these policies can also affect school leaders’ decision-making, teachers’ teaching, and students’ learning. Teachers’ be left feeling dissatisfied, helpless, and worried that more students will give up participating in the competition.

A_4_: *Students’ learning enthusiasm for the Mathematical Olympiad can be affected by China’s education policies. The autonomous enrollment program of universities has been replaced by the “Qiangji Project,” a pilot enrollment plan for colleges. After the new project was implemented, students who got either a gold or silver medal in the CMO are qualified to attend. Even the provincial first prize does not work for many universities’ admissions. As a result, many students lack the motivation to learn.*

In China, the policies related to the five discipline competitions have been reformed in recent years, and many universities’ enrollment policies have changed accordingly. Each university has its own particular field of importance which may also confuse high schools teachers. Overall, however, fewer students can meet the requirements for universities through the Mathematical Olympiad than before, especially when it comes to prestigious universities like Tsinghua University and Peking University.

#### 5.4.2 Cultures

Culture exerts an invisible and formative influence on parents, students and school leaders. The exam-oriented social culture directly affects teachers’ teaching methods, leading teachers to pay too much attention to imparting knowledge while ignoring the process of producing knowledge.

A_2_: *How do mathematicians think about these mathematical problems? How long have they thought this way? What kind of investigation did they do? These thoughts are very inspiring to our study. However, there is no history of mathematics in traditional compulsory education in China, which leads to a problem: in middle school, some teachers always explain a theorem according to the definition provided in books, but they do not know where the theorem originates, so it is difficult for them to arouse students’ interest.*

In China, teaching is a career that requires dedication but does not pay well. In Chinese traditional culture, there is a Confucian slogan regarding education, which states that officialdom is the natural position for good scholars. As a result, if a student graduates from a famous university and pursues a career as a teacher, he or she will experience pressure in terms of public opinion, because the people around them will have higher career expectations of them than simply becoming an ordinary teacher.

A_3_: *I work as a teacher who trains students for the Mathematical Olympiad. My parents are fairly supportive of me, but some of my relatives and friends believe that, because I graduated from the University of Science and Technology of China and studied mathematics for many years, I should have chosen a promising career that would quickly pay well, rather than having chosen to become a teacher.*

Examination culture, as a kind of cultural genre of Chinese, immediately influences teachers’ working style, because students’ grades are directly linked to teachers’ performances. In addition, the internal pursuit of justice and the external one-sided evaluation orientation that students who achieve high marks are excellent in our education and examination culture, stimulate students and parents to pay attention to and enthusiasm for examination. However, at the same time, they cause utilitarian and one-sided problems, such as focusing only on grades or further enrollment. Such problems will gradually react upon the teachers through the penetration of students and parents.

### 5.5 Chronosystem

Overall, 17 participants made 26 negative comments involving their chronosystem. The participants’ teaching experience in the Mathematical Olympiad ranged from 2 years to 30 years, and they have experienced different students, parents, colleagues, and school leaders. Some teachers have even worked in several different cities. However, despite these differences, all the teachers expressed increases in pressure and negative effects on their physical and mental health.

Teachers do the same things to cope with different emotions at different points throughout their teaching careers. One factor that leads to significant emotional distress is the increasingly fierce competition among high schools, which leads to heavier workloads and longer hours for teachers. As time passes, this high pressure and intense workload will lead to exhaustion and unhealthy conditions.

A_19_: *We may have health problems, such as stomach issues, due to great stress. When I return home after class, it is usually after 10 o ‘clock. My brain is always awake after teaching, so I cannot sleep well, which may lead to health problems in the long run.*

With the popularization of the Mathematical Olympiad, the competition among teachers has become more and more fierce; this manifests itself in two main ways. One is that more schools are teaching the Mathematical Olympiad, which results in higher numbers of participating students; the others are the increasingly difficult Mathematical Olympiad problems and longer working hours.

A_20_: *In the year 2000, I worked in a school with many excellent students. At that time, whether I taught them or not, as long as we sent the best students to participate in the competition, they could often get the National First Prize. Students were able to make the top 40 in the province, even though they did not generally receive extra help. However, a private school started achieving good results in the Mathematical Olympiad all of a sudden. At that time, I wondered why. Some schools in Hunan and Hubei had a professional teaching model. They started early, especially Wugang No. 3 Middle School in the Hubei province. Also, Hunan Normal University High school had a group of teachers teaching the Mathematical Olympiad whose specialization was similar to what we provide to students today.*

As time passes, teachers get older and their physical health deteriorates. However, increases in their workload and working hours can exacerbate that change.

### 5.6 Summary of data analysis

This research draws on Kyriacou’s (2001) research, where teachers’ workplace stress is defined as the experience of unpleasant, negative emotions such as anger, tension, frustration, or depression. These emotions can be characterized as a response of negative effects. The research results show that the greatest influences on these teachers’ stress come from the mesosystem and microsystem levels, and that the key players are students and school leaders. Educational policy and culture are found to be key factors from the macrosystem. Overall, long-term stress was seen to affect both teachers’ moods and their physical health. Table 2 offers the main reasons why teachers have negative work-related feelings based on the analysis of the 33 participants and Bronfenbrenner’s ecosystem framework. Some comments may contain multiple sub-factors, so the values given in each of the constructs may not match Table 2. For counting purposes, each such comment is only counted as 1 item. Furthermore, when counting the total number of teachers involved in a given theme, if a single teacher is associated with multiple sub-factors, he is still only counted as 1 item.

**Table 2:** Main factors in teachers’ negative feelings.

Theme	Factors	Sub-factors	Frequency	Number of teachers
Microsystem	Family members	They lack time to spend with family members.	14	11
		Families complain.	3	3
	Teaching tasks	They doubt whether their courses are helpful to students.	6	3
		Excessive workload.	20	17
		Extremely difficult Mathematical Olympiad problems.	22	12
		Long working hours.	29	21
		The need to improve their own abilities.	9	9
Mesosystem	Students	They expect students to get good results.	8	4
		Students give up.	11	10
		Students are success-oriented.	5	3
		Students cannot reach their goals	25	19
		Less gifted students	11	10
	School Leaders	School leaders overly emphasize scores.	15	10
		Leaders do not support teachers	9	8
		Policies in schools are adverse to teachers’ success.	16	13
	Colleagues	There are conflicts between colleagues	14	10
		Lack of professional teaching teams.	6	5
Exosystem	Students’ Parents	Students’ parents do not support the program	18	14
		Students’ parents are overly achievement-oriented	10	9
	Organizations	There is interference from tutoring institutions	10	6
		There is competition among high schools	20	13
Macrosystem	Policies	Policies can affect leaders	5	4
		Policies can affect students	27	14
		Policies can affect teachers	13	12
	Cultures	Cultures can affect teachers	4	3
Chronosystem	Changes in factors	Both their mood at work and their health will worsen.	26	17

In the microsystem, 17 items are related to family members, and 75 items are related to teaching tasks. The total number of teachers involved in this theme is 31. Among the 17 items concerning family members, 14 items suggest teachers’ lack of time to accompany their families, and 3 suggest complaints from teachers’ families, which shows a great impact on teachers’ family life. Teachers’ lack of time to spend with their families is related to their heavy teaching tasks. Among the 75 items concerning teaching tasks, 29 suggest too long working hours, 22 suggest difficult mathematical Olympic problems, and 20 suggest too heavy workload. The frequency of these three statements is rather high. Teachers of mathematically gifted students have relatively long working hours, including breaks like weekends, summer and winter vocations, and weeknights. Working for long hours leaves them almost no rest time throughout the year, severely squeezing the time for personal life. Besides long working hours, they also have a heavy workload. Most of them not only have to complete math teaching of regular classes, but also have to deal with matters related to teaching the Mathematical Olympiad, including searching for competition materials, finishing the exercise, preparing for extra lesson, managing the daily routine of mathematically gifted students, arranging students to go out to study, etc. These complicated affairs constitute the daily work of teachers, and have a relatively high requirements on their physical consumption and professional level. Moreover, as the teaching content of the Mathematical Olympiad is difficult, some teachers can even not understand it. As a result, there are 6 items related to teachers’ suspicion of whether their courses are helpful to students, and 9 items related to teachers’ desire to improve themselves, which shows the anxiety of doubting self-ability, the pain of preparing for too many lessons, the frustration caused by too difficult exercises, and the confusion of lacking guidance. Such emotional drain will also drive teachers to improve themselves.

In the mesosystem, there are 59 items concerning students, 40 concerning school leaders, and 20 concerning colleagues. The total number of teachers involved in this theme is 32. Students are the most important object of teachers’ work, and the reason why some teachers choose to work on mathematics competition may also be students. Teachers of mathematically gifted all expect that their students can get officially-recognized outstanding competition scores, because students’ excellent results can bring teachers a sense of professional identity and belonging. Besides, students’ awards can also bring teachers honors and bonuses. For example, some teachers are granted the title of “Outstanding Coach of the NSHSMC”; a small number of teachers with extraordinary teaching achievements in competitions are transferred to work as administrative leaders of schools; some schools set high bonuses for competitions. At the same time, teachers of mathematically gifted students spend a lot of time and energy on students, hoping that they can achieve better results in the future competition. Hence, the students’ performance has brought great stress to teachers. Twenty-five items are related to students’ failure to achieve good results in competitions, and 8 items show teachers’ expectation that students can achieve good results. Moreover, when students are not talented enough, or students quit halfway, teachers will also be bothered. There are 11 items, respectively, related to these two points of view. Students’ achievements are not only directly linked to teachers’ own interests, but also influence the opinions of school leaders and colleagues towards the teachers of mathematically gifted students. School leaders are the key factor that influences teachers’ work, because their decisions immediately affect the conduct of the teaching activities and the formulation of policies in school. Some leaders have set demands on students’ achievements and have made too many administrative interventions, which has caused teachers much trouble and stress. What involves leaders most is that school policies are not friendly to teachers, and that leaders attach too much importance to competition results, with 16 items and 11 items, respectively. Among those items, there is one point mentioned repetitively by many teachers, that is, the lesson fee for competition teaching is the same as that for regular teaching, which also indicates that the school policy has no obvious inclination to mathematics competition. Among the 20 items concerning colleagues, 14 are related to conflicts between colleagues. Uncooperative colleagues will increase teachers’ workload, and even cause internal friction at work. There are also 6 items concerning the professional teams. Some schools do not have many teachers of mathematically gifted students, so the teachers work alone and lack the professional teams. Therefore, they also hope that the school can set up a professional team to improve teaching efficiency.

In the exosystem, 26 items are related to parents, while 30 items are related to tutoring institutions and competition among schools. The total number of teachers involved in this theme is 22. As an important logistical support, parents’ attitudes towards math competition will directly affect children’s learning, and thus influence teachers’ work to different degrees. Eighteen items suggest that parents do not support participation in math competition, and 10 items indicate that parents are too utilitarian. Parents do not support math competitions mainly because they think that students waste too much time in it, and this will affect their scores of the Gaokao. While a few parents think that competition training costs a lot of money, for which teachers consider parents to be utilitarian. In China, most of the mathematically gifted students have the experience of going to tutoring institutions. This may be recommended by school teachers or arranged by parents. Some teachers do not have the time and energy to study the Mathematical Olympiad knowledge thoroughly, so they think tutoring institutions can help students finish this part of learning, but this influences teachers’ teaching arrangements to some extent. 20 items are related to competition among schools, far more than the 10 items concerning tutoring institutions. In recent years, because of the role discipline competitions play in further enrollment, competition among schools has become more and more fierce, and the most direct manifestation is the competition for sources of students and faculty. Some super schools’ monopolization of sources of students will cause relatively remote schools to fail to recruit quality students. Competition among schools will lead to an increasing workload for teachers. These will all bring stress to teachers, and affect their physical and mental health.

In the macrosystem, most are related to policy, with 41 items suggesting so. The total number of teachers involved in this theme is 19. Educational policies related to competitions will directly affect students’ enrollment, so they have a great impact on students, with 27 items indicating so. Admission policies, such as the previous escort policy, the current trinity system and strengthening foundation plan, will influence students’ initiative for mathematics competitions, school leaders’ view towards the positioning of math competition and the teaching arrangements, all of which will directly affect teachers’ work. In the chronosystem, 26 items indicate that teachers’ physical and mental health are affected.

## 6 Discussion

In China, the Mathematical Olympiad is an important part of mathematics education for the gifted and talented. China’s outstanding achievements in IMO are inseparable from the contributions of frontline teachers (Xiong and Jiang, 2021). Teaching the Mathematical Olympiad, however, puts significant strain on teachers’ personal lives and affects their working hours. In many schools, these teachers also have to teach regular courses. It is common for teachers to quit after several years of teaching the Mathematical Olympiad and only teach regular courses; this may be due to the heavy workload and long hours. The participants in this study are all teachers who teach the Mathematical Olympiad in China. In-depth study of them and digging out the main factors that cause the negative emotions of them will help provide insights for their physical and mental health and professional development.

Using the analysis of Bronfenbrenner’s ecosystem framework, this study found that the main sources of teachers’ stress are usually students, family members, leaders, colleagues, and students’ parents, which is in line with previous studies (Kelchtermans, 2017). Key social factors include other schools, tutoring institutions, educational policies, and cultures. Although the theoretical model is divided into five dimensions, factors are interrelated; as such, we regard them as a whole in order to make the structure more logical.

First of all, it is embodied in the teaching object. Students, of course, have the most effect on teachers. Their behavior problems and lower academic achievement can potentially affect their teachers’ work (Shen et al., 2015; Herman et al., 2018). Different from ordinary students, the teachers of the mathematically gifted face students with high mathematical talents. The math aptitude of these students and the math ability they demonstrate will basically surpass their math teachers at some stage. In the face of students with higher math levels than theirs’, it is normal for teachers to feel stressful. They will doubt whether their teaching is helpful to students, and such stress can become a motivation for them to seek for ways to improve themselves, just as A_18_ mentioned. In addition to the stress on teachers, excellent students will also lead teachers to self-development, and teachers’ ability will also be enhanced during the process of teaching to meet the learning and emotional needs of excellent students. Students’ performance will bring teachers pressure, but it is also the most important criteria for evaluating their teaching. However, this evaluation standard for teachers can also cause anxiety (Ryan et al., 2017). It may even lead to teachers’ frustration and self-doubt in their pursuit of such high standards, thus leading to their resignation (Kelchtermans, 2017). In schools, such test-based accountability policies are generally related to leaders; they are specific measures for managing teachers and reflect the leaders’ attitudes toward the Mathematical Olympiad. Such interference will affect teachers’ enthusiasm, and excessive work pressure will cause them to quit (Ryan et al., 2017). As A_22_ and A_31_ mentioned, the leaders of the school place excessive emphasis on the performance in mathematics competitions. Besides evaluating teachers’ teaching, the results of mathematics competitions are also directly related to students’ further enrollment. Under the guidance of such admission system, Chinese students are overly success-oriented because they usually want to achieve their life goals through diligent study (Li, 2005), and have a strong external motivation to strive for a promising future (Leung, 2001; Wong et al., 2012). However, for various reasons, some students may give up midway through mathematics competitions due to difficulties in adapting, as A_4_ mentioned. Parents, who play the most important roles in students’ family education, are also the important supporters, who are closely related to students’ academic performance. However, some teachers felt that parents’ excessive desires for their children’s success negatively affect learning. This parental mentality is also caused by the overall social environment – the ‘examination culture’ (Wong et al., 2012), showing the essential role that examinations play in China, As A_11_ stated, parents only support exams that are beneficial for students’ further education. Success can bring happiness to families while failures bring shame (Li, 2002). Influenced by this atmosphere, Chinese parents tend to attribute students’ success to their diligence (Leung, 2001). Of course, if there are only a few students who are suitable to learn competitions, teachers of various subject competitions have to compete for the students. The allocation of school resources also affects teachers’ moods; teachers with fewer resources experience greater pressure (Herman et al., 2018). In this study specifically, teachers of various discipline competitions were seen to compete for excellent students.

Secondly, it is embodied in teaching content. The teaching content of mathematics competition mainly includes two parts. The first part is the knowledge of college entrance examination, including sets, functions, trigonometry, progression, etc. All math teachers need to teach this part of knowledge in school. The second part is the knowledge of the Math Olympiad, including geometry, algebra, number theory and combinatorics. Content of these 4 areas only requires teachers of mathematically gifted students to teach, and no requirements are made of other teachers. A problem may involve two or more of these areas. The problems must be solvable by elementary methods, although they may be very difficult. The most important assets in tackling these problems are ingenuity, creativity, and problem-solving skills. In general (with the exception mentioned below), there is not a prescribed list of topics, results, or methods to be mastered by contestants to succeed (Nieto-Said and Sánchez-Lamoneda, 2022). Moreover, most teachers are not familiar with the content in these four areas because they have only studied fragmented knowledge during their university education and have not received systematic training. This poses certain challenges to their work, as was mentioned by Teachers A_5_, A_24_, and A_16_. Consequently, some teachers need to independently study the content before delivering a lesson, which increases their workload in terms of lesson preparation. In addition to the rather unfamiliar teaching content of the Mathematical Olympiad, there are many difficult exercises, so many teachers cannot finish the exercises independently. They need to consult the answers, but sometimes the problem-solving process of the answer is not so ideal. Because the teaching content is difficult, and teachers themselves are not so familiar with it, they may also doubt whether their teaching is effective, and of course sometimes they may choose to let students analyze the exercise. As is also the case in some African countries, teachers are unlikely to put effort into supporting activities that are not curriculum based, and the communication of the importance and relevance of a competition is not clear, so a special school or institute must be established to dedicate to the enhancement of mathematical studies (Baker et al., 2022). In China, there are preliminary math competitions in each province, and annually there are the NSHSMC, the CMO and so on, which produce a large number of test questions every year. Teachers not only need to give lessons, but also should prepare new test questions for students. They themselves also have to bite the bullet and get the questions done, and then explain the question-solving process to the students. Compared with the vivid routine teaching, the teaching process of the Mathematical Olympiad is rather plain. Some teachers just analyze the exercises in class, with each lesson composed of around 10 questions, and their main task when preparing for lessons is to seek for exercises. The unfamiliar teaching content, difficult questions that teachers cannot even solve, and lots of preparation of new questions all make the work of teachers of mathematically gifted students more difficult, so most schools do not have the professional teams. There are mainly two reasons. One is that some teachers are not competent to teach knowledge concerning the Mathematical Olympiad, and the other one is that some teachers are not willing to do so. As the teaching content of the Mathematical Olympiad in school is limited, some students will go to the tutoring institutions as a supplement of on-campus learning.

The Mathematical Olympiad can be used as an after-school learning resource for math-gifted students (Leikin, 2010). Many tutoring institutions in China provide students with preparatory courses for the Mathematical Olympiad. Such institutions are usually in big cities, and students in remote areas have few opportunities to participate, resulting in the uneven distribution of educational resources. Although some teachers felt that such resources could supplement in-class learning, others thought that it interfered with their teaching and created many unnecessary challenges (Liang, 2014).

Finally, it is embodied in teaching time. Different from regular teaching, the teaching time of the Mathematical Olympiad mainly concentrates on weekday evenings, weekends, winter and summer holidays, and other holiday time, which makes the teachers of mathematically gifted students hardly have much time to rest, just as A_14_ mentioned. They need to conduct regular college entrance examination teaching on weekdays, and the Mathematical Olympiad teaching in the rest time, which means they almost work every day. An important reason why teachers work for so long hours is the competition among high schools, just as A_8_ mentioned. More than a decade ago, when competition in math contests was less intense than it is now, students at some school could even win prizes without receiving extra tutoring. However, nowadays, in addition to this influence of tutoring institutions, competition among schools is also intensifying. In an overly competitive environment, many schools will put test scores first. They try to achieve strong test rankings to improve their status in the area, which, in turn, will attract stronger students and increase the school’s reputation (Kai, 2012). To achieve this, school leaders will take measures that give teachers more work and longer hours, thus reducing their job satisfaction. Private schools also make the competition more intense; many teachers said that private schools poach excellent students, which only increases students’ living expenses and does not improve their academic performance (Andersen and Serritzlew, 2007). As in many African countries, top performing schools will push their students to take part in mathematical competitions, and school websites will boast of past performances in competitions (Baker et al., 2022). In addition to the factors discussed above, excessively long work hours and heavy workloads will cause anxiety for teachers (Ferguson et al., 2012; Skaalvik and Skaalvik, 2015). Some teachers even said that they had no time to plan their lessons. In addition to the teaching time, teachers also need to take students to mathematical activities during holidays. For instance, many provinces organize mathematics summer camps and invite university professors, senior teachers, and trainers to teach the participating students(He et al., 2022). Finally, work takes up too much of teachers’ private time, which affects their family lives. Some teachers said that they have no time to spend with their families; this contradiction between work and family life further increases burnout (Liu and Cheung, 2015).

Certainly, over these years the work pressure of teachers has become greater, which is also related to the overall environment. The effects of math competition are controversial. For example, competitions at school level convey an inadequate impression that mathematics is a collection of problems, which means competitions cultivate the ability to answer questions and tasks posed by others (for instance, by the jury), but actually it is very important to ask questions that are relevant to the object studied and can be answered with existing knowledge (Kenderov, 2022). The participants also indicated that the current social environment does not significantly recognize the Mathematical Olympiad. China is also constantly seeking out, selecting, and training top-notch students. As A4 mentioned, policy changes also affect students’ motivation to learn. The current “Qiangji Plan,” which is a plan for strengthening basic disciplines, emphasizes the comprehensive evaluation of students (Du, 2021). At the same time, although the autonomous enrollment program of universities continues in special enrollment policies, including comprehensive evaluation enrollment and direct admission (Du, 2021), the number of students who are directly recommended through the Mathematical Olympiad is decreasing. In this situation, parents will focus more on the Gaokao, and students’ enthusiasm for the Mathematical Olympiad will decrease in order to focus on their overall scores. In some schools, the numbers of Mathematical Olympiad participants are getting smaller and smaller.

## 7 Research conclusion and suggestions

This study explored the stressors of 33 Chinese teachers of the mathematically gifted and the factors that contribute to their stress. Studying the sources of their stress can explain their working conditions, and ultimately improve their work efficiency. These results are also beneficial for education policy and school management, especially in terms of the training and management of teachers of the mathematically gifted and gifted education in general. In this study, the teachers mentioned the most emotions at the mesosystem level, followed by the microsystem, the exosystem, the macrosystem, and the chronosystem. This result is different from most previous relevant studies, such as the work of Cross and Hong (2012) and Chen (2017, 2020) which found the highest proportion of positive emotions are at the nearest microsystem. At the mesosystem level, teachers’ stressors mainly came from students’ learning attitudes and scores and school leaders’ attitudes. In addition, teachers were plagued by conflicts and lack of cooperation with colleagues. At the microsystem level, heavy teaching loads were the main sources of stress; students’ performance and teachers’ families were also seen to affect their work. Factors from the exosystem and macrosystem levels included parents’ attitudes, after-school tutoring institutions, competition among schools, and education policies. Among them, education policies are a key factor because they can affect students’ learning, school leaders’ decision-making, parents’ cognition, and even the operation of training institutions. Finally, at different stages at the chronosystem level, teachers’ moods and physical condition became worse and worse under the long-term effects of the preceding factors. Some teachers even became emotionally numb or suffered from diseases. The most obvious factors that changed over time at different stages were educational policies and competition between schools. Based on this research, both the government and schools should pay attention to the professional development and physical and mental health of teachers of the mathematically gifted. For the teachers themselves, the teaching tasks bring them huge work pressure, so the work efficiency of teachers can be enhanced by improving their professionalism. For teachers’ work environment, a reasonable evaluation system helps reduce teachers’ work stress, so that they can work for their interests. Schools should also provide logistical support for teachers, such as providing teaching resources, establishing a professional teaching team for the Mathematical Olympiad, and supporting student participation in mathematics Olympic activities, so as to improve teachers’ work efficiency. For the whole social environment, the way of selecting students should be scientific and diversified, and the government should also protect some schools from losing talented student, so that teachers in each school can work pleasantly. Based on the conclusions of this study, some suggestions are given as follow.

Firstly, the government and schools should pay attention to teachers’ professional development. From the data collected at the Microsystem level, it is evident that due to the inherent complexity of mathematical Olympiad problems, 12 teachers mentioned that the mathematics competition questions themselves were excessively difficult. Moreover, 9 teachers demonstrated an urgent need to acquire knowledge related to mathematical competitions. Additionally, 3 teachers experienced self-doubt and confusion regarding the effectiveness of their own teaching in facilitating student learning. Above all, the school should build a professional development program to inspire teachers, set up a Mathematical Olympiad team, develop school-based textbooks, promote exchange and cooperation between teachers. Next, schools should improve their facilities and provide resource support for cultivating the gifted students. This could involve, for example, special mathematics classrooms, books, and periodicals for both students and teachers study. At the same time, the government should organize training programs for teachers according to their development needs; schools could also invite mathematicians and mathematics educators to hold lectures related to the cultivation of mathematically gifted students. Finally, the government should focus on the teachers’ learning resources, provide books on the Mathematical Olympiad that meet teachers’ needs and teaching requirements and are professional, readable, and instructive.

Secondly, school leaders should create comfortable working environment for teachers of the mathematically gifted. They should amend the current standard such that that students’ achievements are no longer regarded as criteria for evaluating teachers’ work. Based on the data from the Microsystem and Mesosystem dimensions, it is evident that the workload is perceived as excessively demanding by 17 teachers. Additionally, 11 teachers expressed a lack of time to spend with their families. Furthermore, 8 teachers indicated that they lacked support from their leadership regarding mathematical competitions, while some teachers mentioned that the leadership excessively prioritized students’ competition results, and the school’s evaluation system did not favor their work. School leaders should establish a scientific evaluation standard centered on students’ development, break the teaching barriers of valuing achievement rather than development, and construct a multi-dimensional evaluation mechanism to activate teachers’ enthusiasm. Schools should also fully understand, respect and support the teachers of the mathematically gifted; this could be achieved through managing the relationships between the teachers, students’ parents, and other teachers. Administrators should also seek out the opinions of the teachers regarding the teaching arrangements of the Mathematical Olympiad. Next, Schools should also highlight the successes of both the mathematically gifted students and their teachers to increase morale. Finally, schools could also take a series of specific measures to improve the physical and mental health of teachers, such as organizing regular physical examinations and travel-based activities.

Thirdly, the government should create a good external environment for schools. From the perspectives of the Exosystem and Macrosystem, competition among high schools, interference from tutoring institutions, and competition-related admission policies all affect the teaching work of teachers. First of all, in terms of entrance to university through the Mathematical Olympiad, the education department should establish a scientific selection and elimination mechanism that focuses on examining the students’ comprehensive potential and academic interests and selecting mathematically gifted students through multiple methods. Next, in terms of sourcing students, the education department should clarify the geographical scope of enrollment of each school, standardize the enrollment system, increase the punishment for cross-district illegal enrollment, and strictly supervise its implementation. Finally, a long-term governance mechanism for off-campus tutoring institutions should be established to promote their training, homework, and assessment systems in order to meet the needs of the mathematics competition students, provide professional guidance for teachers of the mathematically gifted, and supplement the teaching resources of public schools.

### 7.1 Limitations and prospects of the study

First of all, the participants were teachers of the mathematically gifted from China. Due to this limited sample size, the universality of the research conclusion is weak (Yin, 2017). As per the ecological system theoretical model, this study counted the number of teachers’ comments in various dimensions, but did not rank the importance of each factor. Future research could use the qualitative data of this study to develop a stress scale for teachers of the mathematically gifted based on the ecological system’s five dimensions, and then rank the importance of the various factors. Secondly, when participants are interviewed, much of the content involves their recall and reflection on past experiences, which may be distorted. Additionally, it is obviously impractical to observe the teaching processes of these teachers throughout their careers, which is why qualitative research are an important means of teacher career development research. Therefore, in the future, more data could be obtained by combining questionnaires with classroom observations when studying teachers of the mathematically gifted. This research could also be combined with other perspectives, such as those of students, school leaders, or colleagues, to evaluate the work of teachers of the mathematically gifted more objectively.

## Data availability statement

The raw data supporting the conclusions of this article will be made available by the authors, without undue reservation.

## Ethics statement

The studies involving humans were approved by University Committee on Human Research Protection of East China Normal University. The studies were conducted in accordance with the local legislation and institutional requirements. The participants provided their written informed consent to participate in this study. Written informed consent was obtained from the individual(s) for the publication of any potentially identifiable images or data included in this article.

## Author contributions

SL: Writing – review & editing, Writing – original draft, Methodology, Investigation, Data curation. YH: Writing – review & editing, Supervision, Project administration, Investigation, Data curation. BX: Writing – review & editing, Supervision, Project administration, Funding acquisition. YW: Writing – review & editing, Investigation, Data curation.
